# Obesity in the United States – Dysbiosis from Exposure to Low-Dose Antibiotics?

**DOI:** 10.3389/fpubh.2013.00069

**Published:** 2013-12-19

**Authors:** Lee W. Riley, Eva Raphael, Eduardo Faerstein

**Affiliations:** ^1^Division of Infectious Disease and Vaccinology, School of Public Health, University of California, Berkeley, CA, USA; ^2^Department of Epidemiology, Institute of Social Medicine, State University of Rio de Janeiro, Rio de Janeiro, Brazil

**Keywords:** obesity, antibiotic residues, intestinal microbiota, food chain, CAFOs, animal husbandry, polysaccharide diet

## Abstract

The rapid increase in obesity prevalence in the United States in the last 20 years is unprecedented and not well explained. Here, we explore a hypothesis that the obesity epidemic may be driven by population-wide chronic exposures to low-residue antibiotics that have increasingly entered the American food chain over the same time period. We propose this hypothesis based on two recent bodies of published reports – (1) those that provide evidence for the spread of antibiotics into the American food chain, and (2) those that examine the relationship between the gut microbiota and body physiology. The livestock use of antimicrobial agents has sharply increased in the US over the same 20-year period of the obesity epidemic, especially with the expansion of intensified livestock production, such as the concentrated animal feeding operations. Observational and experimental studies support the idea that changes in the intestinal microbiota exert a profound effect on body physiology. We propose that chronic exposures to low-residue antimicrobial drugs in food could disrupt the equilibrium state of intestinal microbiota and cause dysbiosis that can contribute to changes in body physiology. The obesity epidemic in the United States may be partly driven by the mass exposure of Americans to food containing low-residue antimicrobial agents. While this hypothesis cannot discount the impact of diet and other factors associated with obesity, we believe studies are warranted to consider this possible driver of the epidemic.

## Introduction

Obesity in non-elderly adults is defined as a body mass index (BMI = weight in kilograms divided by height in meters squared) of 30 or higher (1). Obesity has emerged as an epidemic, especially in the United States, accompanied by a variety of chronic medical problems, including diabetes, hypertension, and dyslipidemia. In the United States, the increase in overweight and obesity prevalence has particularly accelerated in the last 20 years. The overweight prevalence doubled from a mean of 15.1% in 1976–80 to 31.2% in 2001–2004 (2). In 2000, none of the states had an obesity prevalence exceeding 30%; by 2010, 12 states reported an obesity prevalence of >30% (3). Although the prevalence appears to be leveling off, it had increased to 35.7% among adults by 2009–10 (4). A Centers for Disease Control and Prevention (CDC) survey of 3141 counties in the US in 2007 revealed distinct geographic clustering of obesity prevalence, with the highest prevalence (>30.9%) occurring in the Southeastern states and in the Appalachian counties of Tennessee and Kentucky (5).

Many putative causes of obesity and its epidemic in the US have been suggested (6). Most reports attribute the obesity epidemic to factors such as excess food energy intake, changes in diet and eating behavior, and increasing sedentary life style. Undoubtedly, these factors contribute, but can they all account for the rapid increase in this problem that occurred over the last two decades? Recently, a series of experimental and observational studies have described the role and impact of intestinal microbial population structure (microbiota) on body metabolism and energy balance, and how its disruption (dysbiosis) could adversely affect body physiology and health (7–9). If such a mechanism – disruption of the intestinal microbiota – occurred at the population level, it could potentially explain the obesity epidemic.

As with infectious disease epidemics, the obesity epidemic affecting large segments of a population within a short time frame suggests common population-wide exposures. What such common exposures could alter the gut microbiota at the population level? Here, we review a body of literature to support a hypothesis that the American human intestinal microbiota may have been disrupted by chronic, widespread exposures to antimicrobial residues that have increasingly entered our food chain and the environment over the last 20 years. These exposures may be contributing to the obesity epidemic.

Our hypothesis is based on review of relevant literature, performed as follows. We first searched PubMed by cross-referencing the word “obesity” with the following terms: epidemic, prevalence, diet, calorie intake, nutrients, physical activity, lifestyle, host factors, genetics, antibiotics/antimicrobial agents, antibiotic residues in food, antibiotics in plants, antibiotic growth promoters, environmental release of antibiotics, animal husbandry, animal feed, gut microbiome/microbiota, and metagenome. We then found additional references by reviewing the cited references from the primary articles. We excluded abstract reports or conference proceedings. Articles not available electronically were sought at the University of California library collections. The search was limited to publications in English up to August 2013.

## Antibiotics in the Environment and Food

What is the evidence that American food contains antimicrobial agents or that food can become contaminated with them? One major potential source of antibiotics that enter the food chain in the US is the food animal reservoir. The intensification in livestock production and animal feeding operations (AFOs) greatly expanded in the 1990s, which also greatly expanded the therapeutic and prophylactic use of antibiotics (10). In the US, at least 17 classes of antimicrobial agents are approved for use in animal husbandry (11). The estimates of antibiotic use in food animals prior to 2008 are not readily obtainable, but various sources report the use in the US to range from 20.5 million pounds for all purposes in 1999 (12) to 24.6 million pounds a year for non-therapeutic purposes alone (13). An Institute of Medicine report in 1989 estimated that more than 50% of all antimicrobial agents produced in the US are used for livestock (14). The Food and Drug Administration (FDA) reported that in 2011, the sales and distribution of antimicrobial agents approved for use in food-producing animals were 13,542,030 kg (15), which is about four times the total sold and distributed for use to treat human infections (15). Chlortetracycline alone had the highest annual estimated use at 533,973 kg just for swine production in the US (16).

About 75% of antibiotics given to feedlot animals are not absorbed by the body and hence excreted in waste (manure and urine) (17, 18). In 2002, 185 million swine sold in the US generated about 280 million tons of fresh manure; in 2006, chicken produced even more (460 million tons), while, in 2007, beef cattle produced 3.6 million tons of manure (19–21). Environmental pollution from animal waste results from direct discharges, open feedlots, pastures, storage lagoons, stockpiles, and land application fields. Manure is converted into fertilizers and spread over crops. From these sources, contamination can occur on surface water, groundwater, and soil. Based on the amount of antibiotics estimated to be used for growth promotion in animal husbandry, 7.5–18 million pounds of these antimicrobial drugs could be released into the environment annually (12, 13).

In the last 20 years, the above practices as well as the shift from integrated farming operations to concentrated animal feeding operations (CAFOs) have led waste-related pollution to be concentrated in certain geographic regions of the US (18). The largest increase in broiler chicken CAFOs occurred in the Southeastern states in the last 20 years (22) – practically overlapping with the counties with the highest obesity prevalence in the US (5).

Antibiotics released into the environment have varied biodegradability, depending on their chemical property, soil composition, climactic conditions, and other environmental factors (10). Some antibiotics released into soil by manure may be detected up to 5 months (23). Yang and Carlson analyzed tetracyclines and sulfonamides along the Cache la Poudre River in Colorado, and found that while no drugs were found in the pristine, mountain stretch of the river, sulfonamides were found along the remaining stretch and tetracycline concentrations progressively increased downstream from urban areas (24). The tetracycline concentrations correlated with agricultural activity (24). In another study of 139 streams sampled in the US between 1999 and 2000, trimethoprim, erythromycin, lincomycin, and sulfamethoxazole were detected in more than 15% of the samples (25).

Antibiotics can also accumulate in plant tissue. Dolliver et al. showed experimentally that sulfamethazine in manure-amended soil accumulated in corn, lettuce, and potato at concentrations of 0.1–1.2 mg/kg dry weight (26). Others have shown accumulation of low concentrations of antibiotics in carrot roots, green onion, and cabbage (23, 27).

In addition to livestock sources, antimicrobial agents are released into the environment from aquaculture (e.g., shrimp and fish farms) (28–30), spraying fruit orchards and vegetables (31, 32), and from discarded expired drugs, hospital effluents, and other human activities (33). Thus, there is ample evidence that antibiotics can enter our food chain from a variety of sources, and that humans are chronically exposed to these drugs (Table 1). These exposures have greatly increased in the US in the last 20 years, overlapping closely both in time and place with the increasing prevalence of obesity.

**Table 1:** **Food, water, and environmental sources found to contain residues of antimicrobial agents**.

Source	Antimicrobial agents found	Concentrations	Country	Reference
**FOOD**				
Shrimp	Fluoroquinolones	0.1–1 ng/g	USA	(28)
Salmon, trout, shrimp tissues	Fluoroquinolones	0.28–16 ng/g	Canada	(29)
Swine, chicken, shrimp tissues	Fluoroquinolones	1–100 ng/g	China	(34)
Bob veal, heavy calves, heifers, market hogs, non-formula-fed veal, roaster pig, sows	Sulfonamides	0.1–1 ppm	USA	(35)
Bull meat	Moxidectin (milbemycin)	89.13 ppb	USA	(35)
Goat meat	Oxytetracycline	4.66 ppm	USA	(35)
Market hog, roaster pig meat	Carbadox	47–110 ppb	USA	(35)
Catfish, basa	Fluoroquinolones	1.9–6.5 ppb	China	(36)
Honey	Erythromycin	50–1776 ng/g	Turkey	(37)
Corn, green onion, cabbage	Chlortetracycline	2–17 ng/g	USA	(27)
Pig farm waste water	Sulfonamides	20 μg/mL	Vietnam	(38)
Sewage samples	Cefalexin, cefotaxime	>1 μg/mL	Hong Kong, Shenzhen	(39)
Swine farm lagoon	Chlortetracycline	68–1000 μg/L	USA	(40)
Wastewater treatment plant effluent	Minocycline, epitetracycline, tetracycline, doxycycline	95.8–915.3 μg/L	Portugal	(41)
Wastewater treatment plant final effluents	Erythromycin, ciprofloxacin, sulfamethoxazole, tetracycline	0.08, 0.118, 0.243, 0.151 μg/L	Canada	(42)
Wastewater	Chlortetracycline, ciprofloxacin, erythromycin, sulfamethoxazole, tetracycline, trimethoprim	0.69, 0.03–0.14, 0.9–1.7, 0.05–1.9, 0.05–0.85, 0.05–0.71 μg/L	USA	(25, 43)
Cache la Poudre River	Macrolides	0.06–0.17 μg/L	USA	(44)
Wastewater	Sulfamethoxazole	232–9000 ng/L	Austria, Switzerland, USA, Spain, Germany	(45)
Elbe and Saal rivers	Erythromycin, sulfamethoxazole, trimethoprim	30–70, 30–70, <30–40 ng/L	Germany	(46)
Po river	Macrolides	0.7–68.3 ng/L	Italy	(47)
Wastewater treatment plant effluents	Quinolones	40–580 ng/L	France, Italy, Sweden, Greece, Switzerland	(48, 49)
Wastewater treatment plant effluent	Sulfamethoxazole, trimethoprim, ofloxacin, erythromycin	310–400, 180–320, 110 ng/L, 2.5 μg/L	USA, Germany	(50, 51)
Rio Grande river	Sulfamethoxazole	300 ng/L	USA	(51)
Surface water	Erythromycin, sulfamethoxazole	150, 30 ng/L	Germany	(50)
Cattle manure	Chlortetracycline	7.73 mg/L	USA	(52)
Cattle, turkey manure	Monensin	1–4.4, 1.2–1.5 mg/L	USA	(53)
Swine manure	Chlortetracycline	27 mg/L	USA	(54)
Swine slurry	Tetracycline	5–24 mg/L	Germany	(55)
**OTHERS**				
Hospital effluent sludge	Oxofloxacin, ciprofloxacin	0.7–2.0 mg/kg	Sweden	(56)
Hospital effluent	Ciprofloxacin, ampicillin	0.7–124.5, 20–80 μg/L	Germany	(57)
Hospital effluent	Minocycline, epitetracycline, tetracycline, doxycycline	8.1–531.7 μg/L	Portugal	(41)
Hospital effluent	Ciprofloxacin, metronidazole, sulfamethoxazole, trimethoprim, doxycycline	3.6–101, 0.1–90.2, 0.4–12.8, 0.6–7.6, 0.6–7.6 μg/L	Sweden	(58)
Hospital effluent	Sulfamethoxazole, trimethoprim, ofloxacin, ciprofloxacin, lincomycin, penicillin G	400–2100, 2900–5000 ng/L, 25.5–35.5 μg/L, 850–2000, 300–2000, 850–5200 ng/L	USA	(51)
Dairy plant effluent	Lincomycin	700–6600 ng/L	USA	(51)

Of course, we acknowledge that exposure does not necessarily indicate cause and effect, and the evidence to date showing the association is largely ecologic. Furthermore, the impact of changes in dietary intake that also took place in the US in the last 20 years cannot be completely discounted. The average per capita per day energy levels available in the US food supply was 3,400 kcal in 1909–1919, 3,600 kcal in 1990–1999, and 3,900 kcal in 2004 (59). National Center for Health Statistics through series of surveys (National Health Examination Surveys from 1959 to 1970 and the National Health and Nutrition Examination Surveys from 1971 to 2004) shows that the prevalence of overweight and obese Americans increased slowly through the middle of 1970s and then sharply began to rise thereafter (2). Thus, the period of accelerated increase in weight gain overlaps with both increased dietary caloric intake and antibiotic exposures in food. Here, we argue that the effect of diet and exposure to low-residue antibiotics in food on weight gain may be related and that they cannot be easily disassociated. We provide below the biological plausibility evidence for how antibiotics may influence body physiology and how diet contributes to this effect in ways that had not been previously considered.

## Antibiotics as Growth Promoters

In 1946, Moore et al. reported that the administration of low-dose streptomycin and sulfasuxidine in chicken feed caused increased weight gain in chicks (60). Stokstad et al. reported in 1949 that chlortetracycline-containing mash had a growth-promoting effect on poultry (61). In 1950, Luecke et al. reported that streptomycin in combination with vitamin B12 included in a basal diet caused 40% weight gain in pigs (62). These and other similar observations led the food animal industry to gradually adopt the practice of administering subtherapeutic doses of antibiotics as growth promoters and to enhance feed efficiency (63, 64). Debate concerning the economic benefits versus health hazards of this practice began in the 1960s and continues to this day (64–72). Much of the debate has focused on whether or not this practice contributes significantly to the selection of pathogens that cause human drug-resistant infectious diseases. Here, we propose a new hypothesis that this practice may also contribute to the human obesity epidemic.

Several hypotheses to explain the mechanism of weight gain in antibiotic-fed animals have been proposed: (1) suppression of subclinical infections and overt diseases which promotes general health of the animal and hence better nutrition, (2) stimulation of growth of bacteria in the gut that synthesize essential nutrients, (3) suppression of microbes that compete with the host for nutrients, and (4) improvement in intestinal absorption of nutrients (73, 74).

The possibility that humans are exposed to subtherapeutic doses of antibiotics led Ternak to first propose in 2004 that human exposure to low-dose antibiotics may be contributing to weight gain in humans (75). Raoult in 2008 proposed that the gut microbiota modifiers, such as antibiotics and probiotics used in animal husbandry as growth promoters, may contribute to human body weight gain (76). Thus, if humans are indeed chronically exposed to low-dose antibiotics from the environment, there is a good reason to believe that they too can gain weight.

## Change in Intestinal Microbial Population Structure and Its Effect on Body Physiology

A recent series of reports has shown that the structure of the total human intestinal microbial population (microbiota) has a profound influence on body physiology (8, 77–89). The total number of bacterial cells in the human intestine (~100 trillion) is estimated to exceed the total number of somatic and germ cells of the human body by 10-fold (90). Thus, the gut microbiota requires an external supply of energy and nutrients for its own long-term residence in the intestine. It also has to share these nutrients with the human body. In fact, this homeostatic competition, if not disrupted, benefits the host in many different ways. The gut microbiota may even be considered as another vital human organ.

Anaerobic bacterial species that ferment dietary polysaccharides produce short-chain fatty acids (SCFAs) that are taken up by the host as an energy source (91). It is estimated that ~4–10% of the food energy intake of the human body, or 80–200 kcal/day, is derived from these SCFA produced by colonic bacterial fermentation (92, 93). Other bacterial populations produce vitamins (e.g., B and K) and amino acids essential for the host (94). The microbiota also protects the host against pathogens by competitive exclusion (95). Finally, it is needed for the proper development of the intestinal immune system (96, 97). Over a course of tens of thousands of years, the intestinal bacterial population evolved with the *Homo sapiens* body to share and compete for energy and nutrient supply and, in the process, has come to establish a stable equilibrium state. This intestinal microbial equilibrium state contributes to homeostatic maintenance of body weight. A disruption of this steady state (dysbiosis) could affect nutrient and energy utilization by the human body and therefore its physiology.

The human gut microbiota is predominantly composed of four bacterial phyla – Firmicutes, Bacteroidetes, Actinobacteria, and Proteobacteria, with the first two accounting for about 90% of the gut phylotypes (98). Cultivation-independent methods (e.g., 16S rDNA sequence analysis) have revealed that the total number of bacterial species in the gut could be in the range 15,000–36,000 (99). The largest number of bacteria is found in the colon with up to 10^11^ microorganisms/gram of feces (100). A study of 22 samples of intestinal microbiomes from four countries identified three predominant clusters (enterotypes) that differ in species and functional composition, independent of nation or continent (101).

Studies of mammalian host gut microbiota have shown that its alteration can affect body weight (7, 77–79, 81–83, 85). The impact of dysbiosis on obesity and metabolic disorders has been recently reviewed in detail by Harris et al. (9). Backhed et al. first reported that conventionally reared (CONV-R) mice fed polysaccharide-rich diet weighed about 40% more than its germ-free (GF) counterpart fed the same diet (77). When the gut microflora from the CONV-R mice was transferred to the GF mice, the latter underwent a 60% weight gain in 2 weeks (77). The increase in weight of the lean mice was attributed to the gut microflora that allowed energy to be salvaged from otherwise indigestible polysaccharide diet. Ley et al. have shown that obese mice, due to a homozygous mutation in the leptin gene (*ob/ob*), have a 50% reduction in the Bacteroidetes population with a proportional increase in Firmicutes (102). Turnbaugh et al. reported that a transfer of the microbiota from obese mice to GF mice resulted in greater increase in total body fat in the latter (7).

Differences in gut microbiota have also been documented in obese versus lean human subjects (8, 80, 86, 87). One study characterized the fecal microbial communities of obese and lean pairs of adult female monozygotic and dizygotic twins and their mothers and found that obesity was associated with phylum-level changes in the microbiota and reduced bacterial diversity (8). A study by Schwiertz et al. showed that rather than Firmicutes, increased Bacteroidetes to Firmicutes population ratio was associated with obesity (88). Ley et al. followed obese subjects on weight-reducing diet for 1 year and found Bacteroidetes proportion to increase relative to Firmicutes (80). Other authors have observed that weight-reducing diets in obese subjects can alter the gut microbiota species composition, but no major changes in the ratio of Bacteroidetes to Firmicutes between lean and obese subjects were found (84). The study that examined the three enterotypes of human gut microbiota did not find any correlation between Bacteroidetes/Firmicute ratio and BMI (101). Jumpertz et al. prospectively measured ingested and stool calories of lean volunteers, and found that a 20% increase in Firmicutes with a corresponding decrease in Bacteroidetes correlated with an increased energy harvest of ~150 kcal (103). The above studies that compared intestinal microbiomes at the phylum-level show mixed and conflicting results. However, when the intestinal microbiota is examined at the functional group level, correlations with host characteristics begin to be revealed. Several studies have found that obese mice and human subjects have a larger amount of SCFA in their stool than do lean individuals (7, 82, 88). Arumugam et al. reported that while the three human gut enterotypes did not show any correlation with BMI, there was strong correlation between F-type ATPase abundance and increasing BMI (101). Cani et al. showed that lipopolysaccharide purified from *E. coli* injected subcutaneously into mice induced obesity as well as insulin resistance (82).

Another report showed that endotoxin-producing Enterobacter given to GF mice on high-fat diet became obese, while control GF mice fed the same diet did not gain weight (104). Mice given Enterobacter and normal mouse chow did not gain weight. The Enterobacter was obtained from a morbidly obese volunteer subject, whose intestinal microbiota was largely (35%) comprised of this bacterial genus (104). With shift to a diet comprised of whole grain and traditional Chinese medicinal food, the volunteer’s weight decreased >57 kg after 23 weeks, and the Enterobacter population decreased to 1.8% of the intestinal microbiota (104). Indeed, Zhang et al. recently reported that in mice, diet changes explained 57% of the population variation in the intestinal microbiota, whereas genetic mutation accounted for no more than 12% (105).

More recently, Ridaura et al. transplanted fecal microbiota from human monozygotic twins discordant for obesity into GF mice and showed that mice given the microbiota from the obese member of the twin increased in adiposity (89). Then, mice transplanted with fecal microbiota from the co-twins were cohoused. Mice given obese co-twins’ microbiota were prevented from gaining adiposity when cohoused with mice given lean co-twins’ microbiota (89). This transformation was associated with invasion of Bacteroidales from lean into obese microbiota. Mass spectrometry analysis of sera revealed significant increases in branched chain amino acids in mice that received fecal microbiota from obese co-twins, while the microbiota transplanted from lean co-twins exhibited a significantly higher expression of genes involved in plant-derived polysaccharides and a higher concentration of SCFAs (butyrate and propionate)(89). These observations demonstrate that the interactions between diet and gut microbiota can influence body physiology. Such interactions can certainly be profoundly affected by antibiotics.

The next relevant question would be, how does exposure to antibiotics contribute to alteration in intestinal microbial population?

## Evidence of Antibiotic-Induced Changes in the Human Gut Microbiota and Body Physiology

The intestinal microbiota serves as an interface between the human body and its environment. Its perturbation is bound to affect body physiology. We argue that low-dose antibiotics in food are a major contributor to this perturbation. The analysis of the intestinal microbiota or body physiology in humans exposed to antibiotics has been largely confined to those studies of subjects receiving therapeutic drug doses. Blaser and Falkow have suggested that the accelerated disappearance of the normal human microbial community, such as *Helicobacter pylori* in the stomach, due in part to the human use of antibiotics to treat peptic ulcer disease, may be contributing to post-modern conditions, including obesity (106). In a 10-month prospective experimental study of three human subjects given two courses of ciprofloxacin, Dethlefsin and Relman showed that the gut microbiota was rapidly altered with the antimicrobial drug exposure (107). The composition of the microbiota stabilized after 10 months, but in an altered state (107). A study in France of patients with infective endocarditis showed that a 6-week treatment course with vancomycin and gentamicin, but not other antibiotics, was associated with a significant increase in BMI among males >65 years of age (108). Trasande et al. examined the long-term effect of antibiotic exposures in the first 2 years of life of children born in Avon, United Kingdom between 1991 and 1992 (109). They found that exposure to antibiotics during the first 6 months of life, but not during 6–14 or 15–23 months was consistently associated with a later increase in BMI (109).

Thus, even short-term exposures to antibiotics appear to have an effect on BMI in humans. If indeed, a large proportion of Americans are exposed to low-residue antibiotics in the food they eat, chronic exposures to such sources could certainly disrupt the normal steady state gut microbiota, which should be reflected by changes in body physiology that occur at the community and population level. It is interesting to note that adult obesity prevalence increased only slightly from the 1950s through the mid-1970s (involving cohort of people born in the 1930–50s, who would have had no or low-dose exposure to antibiotics in food) (2). The sharp rise began after the 1970s, with the greatest increase occurring after 2000, when obesity among children and teenagers also had the greatest increase (4) (involving cohort of people born during the period of sharpest increase in CAFOs and other forms of intensified livestock production and AFOs). These population-based data do not show a causal effect, but the fact that low-dose antibiotics do cause weight gain in food animals provides a strong rationale to examine the effect of this type of exposure at the human population level.

### Future research

This new hypothesis raises several research questions. To date, the demonstration of the link between intestinal microbiota with changes in body physiology has been experimental, involving mice or selected human subjects. The impact of antibiotic exposures on the gut microbiota ecology and body physiology needs to be examined at the population level. The comparison of gut microbiota of populations residing near CAFOs versus those living away from such places may reveal microbiota population structures associated with differences in body physiology. Antibiotic concentrations in the water and food these populations ingest could be measured for some class of drugs. Serial analysis of the gut microbiome of children at risk for weight gain followed prospectively may reveal changes associated with weight gain. Clearly, obesity has multifactorial causes. Prospective, population-based studies can be conducted to determine the relative proportion of the obesity prevalence that may be contributed by antibiotic exposure in food, if any. Obesity prevalence is also increasing in other countries, especially in the emerging economy nations. The practice and trend of use of antibiotics as growth promoters in animal husbandry can be assessed to examine the relevance of this hypothesis to the obesity epidemic in these other regions of the world. Finally, a more systematic and comprehensive analysis of antimicrobial residues in food and the environment is needed.

## Conclusion

There is accumulating evidence that changes in gut microbiota can affect body physiology. While the exact mechanism remains unsolved, animal experimental and human observation studies provide tantalizing evidence that these changes in the intestine can be triggered by exposures to antimicrobial agents, especially in the context of certain dietary nutrients introduced into the gut, and that these changes can contribute to weight gain. What has been lacking is the epidemiologic evidence in support of this idea. Today, the core gut microbiota of many Americans may be substantially different from that of most Americans living before the 1950s. We propose this idea to be considered to explain part of the obesity epidemic in the US. Clearly more observational as well as experimental studies are necessary to support the hypothesis.

## Conflict of Interest Statement

The authors declare that the research was conducted in the absence of any commercial or financial relationships that could be construed as a potential conflict of interest.
